# Association of Maternal Dietary Patterns With Birth Weight and the Mediation of Gestational Weight Gain: A Prospective Birth Cohort

**DOI:** 10.3389/fnut.2021.782011

**Published:** 2021-11-26

**Authors:** Yan Li, Xuezhen Zhou, Yu Zhang, Chunrong Zhong, Li Huang, Xi Chen, Renjuan Chen, Jiangyue Wu, Qian Li, Guoqiang Sun, Heng Yin, Guoping Xiong, Liping Hao, Nianhong Yang, Xuefeng Yang

**Affiliations:** ^1^Department of Nutrition and Food Hygiene, Hubei Key Laboratory of Food Nutrition and Safety, MOE Key Laboratory of Environment and Health, School of Public Health, Tongji Medical College, Huazhong University of Science and Technology, Wuhan, China; ^2^Hubei Maternal and Child Health Hospital, Wuhan, China; ^3^The Central Hospital of Wuhan, Wuhan, China

**Keywords:** dietary patterns, birth weight (BW), gestational weight gain (GWG), mediation analysis, plant-based foods, pregnant population

## Abstract

The associations among maternal diet, birth weight, and gestational weight gain are still inconclusive. This study aimed to investigate the associations between maternal dietary patterns and birth weight, and further explore whether GWG mediates these associations. A total of 3,334 pregnant women who completed a validated semi-quantitative food frequency questionnaire from the Tongji Maternal and Child Health Cohort were included. Dietary patterns were extracted by using principal component analysis. Regression models and mediation analyses were performed to explore the associations between dietary patterns and birth weight and the effects of GWG on these associations. Five dietary patterns were identified: “Beans-vegetables,” “Fish-meat-eggs,” “Nuts-whole grains,” “Organ-poultry-seafood” and “Rice-wheat-fruits.” Only women following the “Beans-vegetables” pattern had heavier newborns (*β* = 47.39; 95% CI: 12.25, 82.54). Women following the “Beans-vegetables” pattern had significantly lower GWG (*β* = −0.7; 95% CI: −1.15, −0.25) and had a 16% lower risk of excessive GWG and 11% higher odd of adequate GWG. The association between the “Beans-vegetables” pattern and birth weight was negatively mediated by GWG. A dietary pattern enriched in beans and vegetables is beneficial for effectively controlling GWG and increasing birth weight. GWG serves.

**Clinical Trial Registry:** This trial was registered at ClinicalTrials.gov (NCT03099837).

## Introduction

Birth weight is an important index to evaluate the health status of newborns. Inappropriate birth weight is related to an increased risk of infant mortality and diseases in adulthood life (1–4). Therefore, correcting suboptimal birth weight could provide newborns with both short-term and long-term health benefits.

Nutrition plays a crucial role in the growth and development of a fetus. Several studies focusing on specific nutrients and foods have advanced our comprehension of the links between maternal nutrition and birth weight (5). In practice, it has a bias to figure out the exact effect of a single factor due to the interplay between nutrients and foods in the process of digestion and absorption in the human body. Moreover, the specific nutrient deficiency diseases have evolved into chronic conditions which were associated with imbalanced diets over the past few decades (6). To address these, the dietary pattern is applied to characterize the diet on the whole and evaluate its association with birth weight.

It is concordance across many studies that optimal birth weight was associated with what were perceived as healthier diets, such as the Mediterranean diet and the Dietary Approaches to Stop Hypertension diet, which was rich in vegetables, beans, and seafood, among other (7, 8). The pattern rich in vegetables and fruits was associated with lower birth weight in the Norwegian population, whereas women who followed similar diets were more likely to have heavier babies in multiethnic Asian and Chinese populations (9–11). Thus, the relationship between dietary patterns and birth weight is still inconclusive.

Furthermore, gestational weight gain (GWG) of mothers is associated with the birth weight of newborns (12). A meta-analysis revealed that the proportion of excessive GWG was up to 47% according to the Institute of Medicine criteria in 2009 (12). Excessive GWG triggers adverse birth outcomes such as fetal overgrowth and obesity and metabolic dysfunction of offspring for long-term (13, 14). In the context of the association among maternal dietary patterns, GWG and birth weight, we hypothesized that maternal dietary patterns may affect birth weight in two ways: directly or indirectly (mediated by GWG). Therefore, the objective of this study is to figure out the associations between maternal dietary patterns and birth weight, and further explore whether GWG mediates these associations from a large, prospective cohort of pregnant women in central China.

## Materials and Methods

### Study Design

The Tongji Maternal and Child Health Cohort (TMCHC) is a prospective cohort in Wuhan, Hubei province, central China, to investigate the associations between maternal dietary, lifestyle factors, and the pregnant outcomes of mothers and newborns from January 2013 to May 2016. Pregnant women at 8–16 weeks of gestation were enrolled in this cohort. This was the time when they went to the hospital for their first antenatal visit in one of three public hospitals in Wuhan. At enrollment, all participants completed an interviewer-administrated questionnaire which included some baseline information. Trained investigators conducted lifestyle and dietary intake interviews during each trimester. This study was carried out in accordance with the Declaration of Helsinki and approved by the Ethics Review Committee of Tongji Medical College, Huazhong University of Science and Technology. All participants gave informed written consent upon recruitment.

The subjects who were included in this analysis had completed the food frequency questionnaire (FFQ) and been sure to undergo regular prenatal examination and delivery in the above hospitals. Those who reported a previous gestational diabetes mellitus (GDM) or history of diabetes (*n* = 86), multiple gestations (*n* = 135), an implausible total energy intake (<600 or >3,500 kcal/day) (*n* = 81), who had abortion or stillbirth (*n* = 22), or who did not have information about the diagnosis of GDM (*n* = 364) were excluded in this study. Finally, a total of 3,334 women were available (Supplementary Figure 1).

### Dietary Intake Assessment

The dietary intake assessment was obtained with a validated FFQ during the past 4 weeks and all participants completed FFQ during the second trimester of a pregnancy before the diagnosis of GDM (15). This FFQ consisted of food type, frequency of intake, and average consumption per serving of each food in the past 4 weeks (28 days), containing 61 food items and 16 non-overlapping food groups, covering more than 200 kinds of foods (16). We adjusted the 15 categories of frequency into six grades (“never,” “1–3 times per 4 weeks,” “1–3 times per week,” “4–6 times per week,” “1–2 times per day” and “more than two times per day”). The daily intake of each food was calculated by multiplying the number of servings per 4 weeks by the average consumption per serving and then dividing by 28. According to the quantity of energy and nutrient per 100 g of different foods in the China Food Composition Database, energy and nutrient intakes of some food were calculated by this content per 100 g multiplied by daily intake of this food (17). Then we could acquire the daily energy and nutrient intakes by adding up all food intakes. The formulas were as follows:


$$\begin{array}{l} NC\left(unit/d\right)=\overset{n}{\underset{i\;=\;1}{\sum }}\frac{Mi\times Ni\times Pi}{28\times 100} \end{array}$$

Where NC: average daily intake of energy or some nutrient; i: a variety of foods; M: average consumption per serving; N: numbers of servings per 4 weeks; P: quantity of some nutrient in this type of food per 100 g.

### Outcomes Assessments

Gestational weight gain during pregnancy was defined as the difference between the last available weight measurement during pregnancy and the pre-pregnancy weight. The last available weight measurement was measured by investigators on admission to the hospital while awaiting delivery. The pre-pregnancy weight was self-reported using a questionnaire at the time of enrollment during their first antenatal visit to the hospital. Birth weight, birth length, and sex of newborns were obtained through medical records. Some important definitions included: normal birth weight referred to the neonatal birth weight ≥2,500 and <4,000 g; the ponderal index (PI) was calculated as birth weight (kg)/birth length^3^ (m^3^); the newborns were defined as large for gestational age (LGA) when the body weight was >90th percentile for gestational age and small for gestational age (SGA) when it was <10th percentile for gestational age; excessive GWG, adequate GWG, and insufficient GWG were evaluated based on the recommendation of Institute of Medicine (18).

### Covariates Assessment

The demographic and socioeconomic characteristics, anthropometric parameters, and lifestyle of the participants were recorded using a structured questionnaire at the baseline enrollment. The questionnaire was completed by trained investigators, including the information of maternal age, educational level, average personal income, ethnicity, pre-pregnancy weight, height, history of the disease, history of the family disease, parity, physical activity, consumption of smoke and alcohol, and so on. Maternal age was categorized as ≤24, 25–29, 30–35, and ≥36 years old. Ethnicity was categorized as Han Chinese and others. Education level was divided into ≤9 (junior high school or under), 10–12 (senior high school or technical secondary school), 13–15 (bachelor or college degree), and ≥16 (master or above) years (19). Per capita monthly income was divided into five categories: ≤1,000, 1,001–2,999, 3,000–4,999, 5,000–9,999, and ≥ 10,000 CNY. Pre-pregnancy body mass index (BMI) was divided into four categories according to the BMI classification criteria suitable for Chinese people: <18.5, 18.5–23.9, 24–27.9, and ≥28 kg/m^2^. Smoking status was divided into “yes (either smoking or passive smoking)” and “no (none)” according to whether smoking was a habit before pregnancy, or whether participants were frequently exposed to second-hand smoke (more than 15 min a day and more than 3 days a week, lasting half a year). Alcohol consumption was divided into “yes” and “no” categories based on whether alcohol was consumed before pregnancy (more than 3 days a week, lasting half a year). History of disease included chronic non-communicable diseases, infectious disease, and hereditary disease. History of a family disease considered these diseases of immediate families (parents and siblings). The history of specific diseases was considered as binary variables, divided into “yes” and “no”.

### Statistical Analysis

Dietary patterns were extracted by principal component analysis. This analysis was a data-driven technique that reduced the dimensions of the data and grouped correlated variables to identify common factors/components (i.e., dietary patterns) by varimax rotation. The principle of principal component analysis was to decompose the total variance of the original indicators into the sum of the variances of several independent composite factors. The first principal component contributed the most to explaining total variation. The greater the contribution of variation among the factors, the better their ability to combine the original indicators. So, the factors represented the combinations of food consumed by individual participants. The number of factors retained was based on the eigenvalues, the breakpoints of the scree plot, cumulative variances, and factor interpretability. Thereinto, the factor loading reflected the relevance between the original 16 non-overlapping food group frequency and newly extracted factors (dietary patterns). In this study, the food groups with factor loadings of 0.40 or higher on a factor were considered important to the interpretability of each pattern. As the patterns were extracted, each participant had the dietary pattern score corresponding to each dietary pattern. The score was calculated by summing the mean standardized frequency of food groups weighted by their factor loadings. Higher dietary pattern scores indicated greater adherence to the extracted patterns (10, 20).

We described the covariates and dietary characteristics by using ANOVA or independent sample *t*-tests. To explore the associations between dietary pattern scores and pregnant outcomes, we treated dietary pattern scores as quartiles and as continuous variables. Multivariate linear regression models were used to analyze the relations between dietary pattern scores and continuous pregnant outcomes, such as GWG and birth weight. In addition, the changes in pregnancy outcomes and *P*-value were calculated when the dietary pattern score increased by one unit. For the relations between scores and binary pregnant outcomes, such as excessive GWG, adequate GWG, insufficient GWG, SGA, and LGA, Logistic regression models were used. Stratified analyses were conducted by sex of newborns. Mediation analysis was an approach to assess the importance of various pathways and mechanisms, which had expanded over the past decade (21). We used mediation analysis to explore the effect of GWG on the associations between dietary patterns and birth weight. It was performed using bootstrapping, and Model 4 was run in Process (V3.2) with covariates in SPSS (22). All statistical analyses were performed using the SPSS software version 22 (IBM Corp., Armonk, NY). A two-sided α of less than 0.05 was considered statistically significant.

## Results

### Dietary Patterns and Pregnant Outcomes

Overall, 3,334 participants were included in this analysis. Five dietary patterns were identified (Table 1), and we named them “Beans-vegetables,” “Fish-meat-eggs,” “Nuts-whole grains,” “Organ-poultry-seafood” and “Rice-wheat-fruits.” The average age was 28.12 years, and the average pre-pregnancy BMI was 20.77 kg/m^2^. Almost all participants were Han Chinese (97.0%). Other social and demographic characteristics of participants were present in Supplementary Table 1. The scores of each pattern had significant differences in maternal educational level, average personal income, age, parity, and so on, as displayed in Supplementary Table 2.

**Table 1:** Factor loading matrix for dietary patterns identified by principal component analysis (*n* = 3,334)^a^.

**Food groups^b^**	**Dietary patterns**				
	**Beans-vegetables**	**Fish-meat-eggs**	**Nuts-whole grains**	**Organ-poultry-seafood**	**Rice-wheat-fruits**
Root vegetables	0.70	—	—	—	—
Mushrooms and algae	0.58	—	—	—	—
Melon and solanaceous vegetables	0.57	—	—	—	—
Beans and bean products^c^	0.54	—	—	—	—
Leafy and cruciferous vegetables	0.46	—	—	—	—
Red meat	—	0.61	—	—	—
Freshwater fishes	—	0.59	—	—	—
Eggs	—	0.58	—	—	—
Nuts	—	—	0.70	—	—
Whole grains	—	—	0.48	—	—
Dairy products^d^	—	—	0.48	—	—
Animal organ and blood	—	—	—	0.64	—
Seafood	—	—	—	0.64	—
Poultry	—	—	—	0.58	—
Rice and wheat products	—	—	—	—	0.84
Fruits	—	—	—	—	0.51
Cumulative variance explained (%)^e^	11.4	20.7	29.6	38.3	45.1

a *Values were factor loadings (correlation coefficients) between each food frequency variable and the dietary pattern derived from principal component analysis*.

b *Food groups were sorted by the size of loading coefficients. Absolute values <0.4 were not listed for simplicity*.

c *Denoted soybean, mung bean, soybean milk, bean curd, and so on*.

d *Denoted milk, milk powder, and yogurt*.

e *Percentage of variance in total food intake explained by patterns*.

Pregnant outcomes of all participants were presented in Supplementary Table 3. The average GWG was 15.87 ± 4.49 kg and the average birth weight of newborns was 3338.19 ± 448.46 g. For mothers, excessive, adequate, and insufficient GWG made up 44.9, 33.3, and 21.8%, respectively. For newborns, SGA, and LGA accounted for 7.3 and 8.2%, respectively.

### Dietary Patterns in Relation to Normal Birth Weight and Other Birth Outcomes

Whether unadjusted or adjusted, the relationships between dietary pattern scores and normal birth weight only existed in the “Beans-vegetables” pattern, as displayed in Table 2. After adjusted covariates, the newborn birth weight of the highest quartile increased 47.39 g (95% CI: 12.25, 82.54; *P*_trend_ = 0.012). Considering the score as a continuous variable, normal birth weight increased by 17.58 g (95% CI: 5.18, 29.98; *P* = 0.005) with one unit increase in this score. Although “the Fish-meat-eggs” pattern score had no significant relationship with birth weight, the risk of LGA increased 18% as the score increased by one unit (95% CI: 1.04, 1.34). Other associations between dietary pattern scores and birth outcomes were exhibited in Table 3.

**Table 2:** Associations between dietary pattern scores and normal birth weight (g) (*n* =2,847)^a^.

**Dietary patterns**	**Quartiles of dietary pattern scores**				***P*** **for trend**	**Per unit increase**	***P*** **-value**
	**Q1**	**Q2**	**Q3**	**Q4**			
**Beans-vegetables**							
Mean (min, max)	−1.30 (−3.89–0.65)	−0.29 (−0.65, 0.05)	0.37 (0.05, 0.71)	1.22 (0.72, 3.11)			
Model 1	Reference	12.27 (−22.84, 47.34)^b^	13.81 (−21.69, 49.31)	45.40 (10.17, 80.63)	0.015	17.69 (5.27, 30.11)	0.005
Model 2	Reference	12.22 (−22.88, 47.31)	13.53 (−21.99, 49.05)	44.91 (9.66, 80.16)	0.016	17.65 (5.23, 30.08)	0.005
Model 3	Reference	12.97 (−22.05, 27.99)	12.73 (−22.63, 48.10)	47.39 (12.25, 82.54)	0.012	17.58 (5.18, 29.98)	0.005
Boy	Reference	2.26 (−46.91 ,51.43)	12.62 (−36.42 ,61.66)	56.79 (7.80,105.78)	0.024	20.41 (2.91, 37.91)	0.022
Girl	Reference	23.89 (−25.68 ,73.47)	5.5 (−45.40,56.4)	34.63 (−15.78 ,85.05)	0.271	12.80 (−4.70, 30.30)	0.152
**Fish-meat-eggs**							
Mean (min, max)	−1.32 (−4.36, −0.60)	−0.24 (−0.60, 0.07)	0.37 (0.07, 0.68)	1.19 (0.68, 3.12)			
Model 1	Reference	13.52 (−21.74, 48.78)	30.74 (−4.50, 65.97)	15.51 (−19.75, 50.76)	0.263	4.85 (−7.58, 17.28)	0.444
Model 2	Reference	10.08 (−25.28, 45.44)	27.19 (−8.19; 62.56)	15.06 (−20.24, 50.37)	0.275	4.93 (−7.52, 17.37)	0.438
Model 3	Reference	7.37 (−27.88, 42.63)	29.68 (0–5.67, 65.03)	0.39 (−36.00, 36.79)	0.669	1.07 (−11.94, 14.07)	0.842
Boy	Reference	−8.63 (−58.25, 41.00)	35.85 (−14.25, 85.95)	0.62 (−49.79, 51.02)	0.560	4.71 (−13.49, 22.91)	0.612
Girl	Reference	21.13 (−28.92, 71.17)	23.52 (−26.07, 73.12)	−7.54 (−60.21, 45.14)	0.858	−1.29 (−19.81, 17.23)	0.892
**Nuts-whole grains**							
Mean (min, max)	−1.34 (−5.26, −0.65)	−0.25 (−0.65, 0.10)	0.41 (0.10, 0.72)	1.18 (0.72, 2.75)			
Model 1	Reference	42.03 (6.65, 77.40)	18.41 (−16.73, 53.54)	35.29 (0.29, 70.29)	0.142	11.21 (−1.30, 23.73)	0.079
Model 2	Reference	40.35 (4.84, 75.86)	15.25 (−20.14, 50.65)	35.24 (0.25, 70.23)	0.151	11.16 (−1.35, 23.68)	0.080
Model 3	Reference	37.40 (2.06, 72.75)	2.20 (−32.98, 37.38)	18.44 (−17.31, 54.19)	0.732	4.16 (−8.63, 16.95)	0.524
Boy	Reference	27.07 (−21.66, 75.81)	−11.13 (−60.36, 38.10)	7.61 (−43.04, 58.27)	0.840	2.26 (−16.10, 20.63)	0.809
Girl	Reference	47.44 (−4.18, 99.05)	23.88 (−26.35, 74.10)	38.06 (−12.26, 88.37)	0.259	9.13 (−8.80, 27.06)	0.318
**Organ-poultry-seafood**							
Mean (min, max)	−1.25 (−2.56, −0.73)	−0.37 (−0.73, −0.05)	0.30 (−0.04, 0.66)	1.31 (0.66, 3.78)			
Model 1	Reference	−8.44 (−43.86, 26.97)	16.65 (−18.70, 51.99)	−0.17 (−35.53, 35.20)	0.664	2.38 (−10.10, 14.85)	0.709
Model 2	Reference	−8.07 (−43.50, 27.35)	18.35 (−17.00, 53.70)	−0.04 (−35.38, 35.31)	0.642	2.51 (−9.96, 14.97)	0.693
Model 3	Reference	−7.14 (−42.24, 27.97)	24.96 (−10.08, 60.00)	1.84 (−33.56, 37.25)	0.495	4.71 (−7.79, 17.20)	0.460
Boy	Reference	−21.82 (−70.58, 26.94)	24.00 (−25.18, 73.18)	3.75 (−45.40, 52.90)	0.477	8.09 (−9.18, 25.36)	0.358
Girl	Reference	11.20 (−39.34, 61.74)	32.12 (−17.66, 81.89)	0.22 (−50.82, 51.27)	0.785	0.93 (−17.35, 19.21)	0.920
**Rice-wheat-fruits**							
Mean (min, max)	−1.01 (−21.54, −0.23)	−0.01 (−0.23, 0.18)	0.33 (0.18, 0.47)	0.69 (0.47, 1.67)			
Model 1	Reference	8.05 (−27.21, 43.31)	8.45 (−26.67, 43.56)	0.44 (−34.81, 35.68)	0.973	4.49 (−11.41, 20.39)	0.580
Model 2	Reference	−0.71 (−36.64, 35.23)	−1.12 (−37.05, 34.80)	0.12 (−35.19, 35.43)	0.999	4.25 (−11.64, 20.14)	0.600
Model 3	Reference	−6.52 (−42.38, 29.34)	0.84 (−35.06, 36.74)	−2.17 (−37.71, 33.38)	0.986	6.76 (−9.53, 23.05)	0.416
Boy	Reference	−14.70 (−64.99, 35.60)	−20.39 (−70.41, 29.64)	−19.82 (−70.36, 30.72)	0.401	7.60 (−17.17, 32.36)	0.548
Girl	Reference	−2.12 (−53.23, 48.99)	20.19 (−31.61, 71.99)	19.80 (−30.29, 69.88)	0.319	2.95 (−18.54, 24.44)	0.788

a *Multivariate linear regression models were as followed: Model 1, crude model; Model 2, adjusted for other four dietary patterns; Model 3, adjusted for Model 2 + maternal age, physical activity, ethnology, maternal education, average personal income, family history of diabetes, family history of obesity, smoking habit, alcohol habit, parity, pre-pregnancy BMI, GDM, gestational weight gain, and total energy intake*.

b *Values were presented as *β* (95% CI) (all such values)*.

**Table 3:** Associations between maternal dietary pattern scores and pregnant outcomes^a^.

**Pregnant outcomes**	**Dietary patterns**				
	**Beans-vegetables**	**Fish-meat-eggs**	**Nuts-whole grains**	**Organ-poultry-seafood**	**Rice-wheat-fruits**
GWG (kg)^b^	−0.36 (-0.52,−0.20)^**^	0.18 (0.01, 0.34)^*^	0.23 (0.07, 0.39)^*^	−0.01 (-0.17, 0.15)	0.26 (0.04, 0.48)^*^
Birth weight (g)^b^	32.25 (10.40, 54.11)^*^	2.94 (-18.93, 24.79)	3.79 (-18.23, 25.80)	4.30 (-16.46, 25.05)	6.92 (-22.87, 36.71)
Normal birth weight (g)^b^	17.58 (5.18, 29.98)^*^	1.07 (-11.94, 14.07)	4.16 (-8.63, 16.95)	4.71 (-7.79, 17.20)	6.76 (-9.53, 23.05)
Birth length (cm)^b^	0.09 (0.01, 0.16)^*^	−0.04 (-0.11, 0.04)	0.00 (-0.07, 0.08)	−0.02 (-0.09, 0.05)	0.02 (-0.12, 0.09)
Ponderal index (kg/m^3^)^b^	0.15 (0.04, 0.26)^*^	0.07 (-0.04, 0.18)	0.01 (-0.10, 0.12)	0.06 (-0.41, 0.17)	0.06 (-0.09, 0.21)
Excessive GWG^c^	0.84 (0.77, 0.91)^**^	1.07 (0.99. 1.16)	1.07 (0.99, 1.15)	0.94 (0.87, 1.01)	1.01 (0.92, 1.12)
Adequate GWG^c^	1.11 (1.03, 1.21)^*^	1.04 (0.96, 1.13)	1.02 (0.94, 1.11)	1.04 (0.96, 1.12)	1.14 (1.02, 1.26)^*^
Insufficient GWG	1.14 (1.03, 1.26)^*^	0.85 (0.77, 0.95)^*^	0.87 (0.79, 0.96)^*^	1.06 (0.96, 1.17)	0.83 (0.74, 0.93)^*^
SGA^c^	0.89 (0.78, 1.02)	0.97 (0.85, 1.10)	0.95 (0.83, 1.08)	0.98 (0.87, 1.11)	0.97 (0.86, 1.10)
LGA^c^	1.04 (0.92, 1.18)	1.18 (1.04, 1.34)^*^	1.00 (0.88, 1.13)	1.07 (0.95, 1.21)	1.11 (0.98, 1.26)

a *Multivariate linear regression and multinomial logistic regression models were used. Models were adjusted for maternal age, physical activity, ethnology, maternal education, average personal income, family history of diabetes, family history of obesity, smoking habit, alcohol habit, parity, pre-pregnancy BMI, gestational diabetes mellitus status, gestational weight gain, infant sex, total energy intake, and other four dietary patterns. LGA, large for gestational age; SGA, small for gestational age; GWG, gestational weight gain*.

b *Values were presented as *β* (95% CI) for continuous variables*.

c *Values were presented as OR (95% CI) for categorical variables*.

* *P < 0.05*.

** *P < 0.001*.

After stratified analysis according to the offspring sex, the relation of “Beans-vegetables” only appeared in boys. Compared to the lowest quartile, the birth weight of the highest quartile increased 56.79 g (95% CI: 7.8, 105.78; *P*_trend_ = 0.024). One unit increase in dietary pattern score was associated with the increase of 20.41 g of birth weight of boys (95% CI: 2.91, 37.91, *P* = 0.022).

The contributions of nutrients on the association between “Beans-vegetables” patterns and normal birth weight were explored. For total newborns, the association of “Beans-vegetables” pattern score and birth weight was not attenuated substantially after adjusting various nutrients. However, for boys, the association between “Beans-vegetables” pattern score and birth weight for comparisons of highest with lowest quartiles was no longer significant after further adjustment for plant protein (*β* = 50.66; 95% CI: −1.72, 103.04), fiber (*β* = 50.88; 95% CI: −3.36, 105.11), total iron (*β* = 47.65; 95% CI: −4.68, 99.98) and non-heme iron (*β* = 49.79; 95% CI: −2.5, 102.09), which indicated that these nutrients contributed to the association between “Beans-vegetables” pattern score and birth weight (Supplementary Table 4).

### Dietary Patterns in Relation to GWG

Associations between the degree of adherence to the dietary patterns and GWG were exhibited in Table 4. Except for the “Organ-poultry-seafood” pattern, the dietary pattern scores of the other four patterns were either significantly correlated or inversely correlated with GWG. Compared to the lowest quartile of “Beans-vegetables” patterns, the GWG of the highest quartile reduced 0.7 kg (95% CI: −1.15, −0.25; *P*_trend_ = 0.002). With a unit of “Beans-vegetables” pattern score increased, GWG decreased 0.36 kg (95% CI: −0.52, −0.20; *P* < 0.001). In addition, this pattern reduced the 16% risk of excessive GWG and increased the 11% likelihood of adequate GWG (95% CI: 0.77, 0.91 and 95% CI: 1.03, 1.21, respectively), while it increased 14% risk of insufficient GWG in the meantime (95% CI: 1.03, 1.26). For the other three patterns, the GWG of the highest quartile all showed an increase compared with the lowest quartile. GWG increased 0.18 kg for “Fish-meat-eggs” (95% CI: 0.01, 0.34), 0.23 kg for “Nuts-whole grains” (95% CI: 0.07, 0.39) and 0.26 kg for “Rice-wheat-fruits” (95% CI: 0.04, 0.48) as score increased by one unit. And the risks of insufficient GWG for these three patterns decreased 15, 13, and 17%. Moreover, the “Rice-wheat-fruits” pattern increased the 14% likelihood of GWG in the appropriate range (95% CI: 1.02, 1.26) (Table 3).

**Table 4:** Associations between dietary pattern scores and gestational weight gain (kg) (*n* = 3,110)^a^.

**Dietary patterns**	**Quartiles of dietary pattern scores**				***P*** **for trend**	**Per unit increase**	***P*** **-value**
	**Q1**	**Q2**	**Q3**	**Q4**			
**Beans-vegetables**							
Mean (min, max)	−1.30 (−3.89, −0.65)	−0.29 (−0.65, 0.05)	0.37 (0.05, 0.71)	1.22 (0.72, 3.11)			
Model 1	Reference	−0.05 (−0.50, 0.39)^b^	−0.30 (−0.75, 0.15)	−0.60 (−1.05, −0.15)	0.005	−0.31 (−0.47, −0.15)	<0.001
Model 2	Reference	−0.05 (−0.49, 0.39)	−0.34 (−0.78, 0.11)	−0.65 (−1.10, −0.21)	0.002	−0.32 (−0.48, −0.17)	<0.001
Model 3	Reference	−0.13 (−0.57, 0.31)	−0.35 (−0.79, 0.10)	−0.70 (−1.15, −0.25)	0.002	−0.36 (−0.52, −0.20)	<0.001
**Fish-meat-eggs**							
Mean (min, max)	−1.32 (−4.36, −0.60)	−0.24 (−0.60, 0.07)	0.37 (0.07, 0.68)	1.19 (0.68, 3.12)			
Model 1	Reference	0.19 (−0.26, 0.64)	0.10 (−0.35, 0.54)	0.62 (0.17, 1.06)	0.015	0.20 (0.04, 0.36)	0.013
Model 2	Reference	0.25 (−0.20, 0.70)	0.15 (−0.30, 0.59)	0.71 (0.26, 1.15)	0.005	0.23 (0.07, 0.39)	0.004
Model 3	Reference	0.16 (−0.30, 0.60)	0.02 (−0.13, 0.47)	0.60 (0.14, 1.05)	0.028	0.18 (0.01, 0.34)	0.035
**Nuts-whole grains**							
Mean (min, max)	−1.34 (−5.26, −0.65)	−0.25 (−0.65, 0.10)	0.41 (0.10, 0.72)	1.18 (0.72, 2.75)			
Model 1	Reference	0.32 (−0.13, 0.77)	0.66 (0.21, 1.10)	0.65 (0.21, 1.10)	0.001	0.26 (0.10, 0.42)	0.001
Model 2	Reference	0.22 (−0.23, 0.67)	0.54 (0.10, 0.99)	0.65 (0.20, 1.09)	0.002	0.25 (0.09, 0.41)	0.002
Model 3	Reference	0.10 (−0.34, 0.55)	0.45 (0.08, 0.90)	0.55 (0.10, 1.01)	0.006	0.23 (0.07, 0.39)	0.006
**Organ-poultry-seafood**							
Mean (min, max)	−1.25 (−2.56, −0.73)	−0.37 (−0.73, −0.05)	0.30 (−0.04, 0.66)	1.31 (0.66, 3.78)			
Model 1	Reference	−0.25 (−0.70, 0.20)	−0.17 (−0.61, 0.28)	−0.04 (−0.49, 0.41)	0.979	−0.01 (−0.16, 0.15)	0.945
Model 2	Reference	−0.21 (−0.65, 0.24)	−0.14 (−0.58, 0.31)	−0.06 (−0.50, 0.39)	0.886	−0.01 (−0.17, 0.15)	0.890
Model 3	Reference	−0.18 (−0.62, 0.27)	−0.13 (−0.57, 0.31)	0.04 (−0.44, 0.45)	0.929	−0.01 (−0.17, 0.15)	0.934
**Rice-wheat-fruits**							
Mean (min, max)	−0.89 (−6.51, −0.23)	−0.01 (−0.23, 0.18)	0.33 (0.18, 0.47)	0.69 (0.47, 1.67)			
Model 1	Reference	0.78 (0.33, 1.22)	1.05 (0.60, 1.49)	1.14 (0.70, 1.58)	<0.001	0.43 (0.22, 0.64)	<0.001
Model 2	Reference	0.80 (0.35, 1.25)	1.06 (0.60, 1.51)	1.20 (0.76, 1.64)	<0.001	0.44 (0.22, 0.65)	<0.001
Model 3	Reference	0.58 (0.13, 1.04)	0.69 (0.24, 1.15)	0.76 (0.32, 1.21)	<0.001	0.26 (0.04, 0.48)	0.021

a *Multivariate linear regression models were as followed: Model 1, crude model; Model 2, adjusted for other four dietary patterns; Model 3, adjusted for Model 2 + maternal age, physical activity, ethnology, maternal education, average personal income, family history of diabetes, family history of obesity, smoking habit, alcohol habit, parity, pre-pregnancy BMI, GDM, and total energy intake*.

b *Values were presented as *β* (95% CI) (all such values)*.

### Mediation of GWG in the Relationship Between “Beans-Vegetables” Pattern and Normal Birth Weight

Mediation analysis was performed to clarify the role of GWG in the relationship between the “Beans-vegetables” pattern and normal birth weight, as displayed in Table 5. We found that the relationship between the “Beans-vegetables” pattern and birth weight was negatively mediated by GWG (*β*_indirect_ = −4.50; 95% CI: −7.27, −2.04). After adjusted covariates, this mediation was still significant with an estimated mediating proportion of −26% (*β*_indirect_ = −4.67; 95% CI: −7.69, −1.87). Besides, this mediating effect also remained in newborns of boys, and the estimated mediating proportion was −21.3% (*β*_indirect_ = −4.44; 95% CI: −8.68, −0.41).

**Table 5:** Mediating effects of gestational weight gain (kg) (n = 2,793)^a^.

**Variables**	**Direct effect**	**Indirect effect**	**Estimated percent mediated (%)**
Model 1	22.99 (10.68, 35.30)^b^	−4.50 (−7.27, −2.04)	−24.3
Model 2	23.17 (10.84, 35.49)	−4.70 (−7.49, −2.31)	−25.6
Model 3	22.64 (10.44, 34.84)	−4.67 (−7.69, −1.87)	−26.0
Boy	25.29 (8.05, 42.53)	−4.44 (−8.68, −0.41)	−21.3

a *Mediation analyses were used. Models were as followed: Model 1, crude model; Model 2, adjusted for other four dietary patterns; Model 3, adjusted for Model 2 + maternal age, physical activity, ethnology, maternal education, average personal income, family history of diabetes, family history of obesity, smoking habit, alcohol habit, parity, pre-pregnancy BMI, gestational diabetes mellitus, gestational weight gain, and total energy intake*.

b *Values were presented as *β* (95% CI) (all such values)*.

## Discussion

We extracted five dietary patterns to represent the dietary habits of pregnant women in central China, naming them as “Beans-vegetables,” “Fish-meat-eggs,” “Nuts-whole grains,” “Organ-poultry-seafood,” and “Rice-wheat-fruits,” respectively. Women who tended to have higher adherence to the “Beans-vegetables” pattern had infants with relatively high birth weight. Higher adherence to the “Beans-vegetables” pattern was significantly associated with the decrease of GWG, the reduction of excessive GWG risk, and the increase of adequate GWG odd. The association between the “Beans-vegetables” pattern and birth weight was partly mediated by GWG.

In this study, we observed that the “Beans-vegetables” pattern contributed to increasing birth weight in the normal range, without generating the risk of LGA. Consistent with our results, some studies came to the conclusion that maternal dietary patterns rich in plant foods were associated with larger birth sizes in multiethnic Asian and Chinese populations (10, 11). The study of Zulyniak et al. also proved among South Asians living in Canada that a plant-based diet was associated with higher birth weight but not associated with the risk of having an SGA or LGA newborns (23). The “Beans-vegetables” pattern identified in the present study also decreased GWG and controlled it in an optimal range. Similar results existed in other studies. Women from a mother-offspring cohort in Singapore who tended to consume similar plant-based foods had a decreased risk for inadequate GWG and excessive GWG (24). Beyond that, in a prospective cohort of Brazilian pregnant women, the “common-Brazilian” pattern rich in beans had no association with excessive GWG and was positively associated with adiponectin, which had characteristics of anti-obesity and anti-inflammation (25). Thus, a dietary pattern enriched in beans and vegetables has a beneficial effect on optimal birth weight and GWG.

Previous studies have found a positive link between GWG of women and the birth weight of newborns, and GWG above the recommendations was associated with a higher risk of LGA (12, 26, 27). Lu et al. and Wei et al. also found that the effect of a specific dietary pattern on GWG of pregnant women and the birth weight of newborns coexisted in the Chinese Guangzhou population (11, 28). To figure out the potential function of GWG, we further performed a mediation analysis to clarify the role of GWG in the relationship between the “Beans-vegetables” pattern and birth weight. As mentioned above, considering GWG, both the total effect and the direct effect of the “Beans-vegetables” pattern were positive on birth weight, while the indirect effect was negative. As a result, this indirect effect weakened the total effect. GWG may serve as a mediator in the association of the “Beans-vegetables” pattern and birth weight. That means, on one hand, the “Beans-vegetables” pattern itself could directly promote the increase of birth weight, on the other hand, it may also indirectly prevent excess birth weight by controlling GWG.

Our further investigation revealed that plant protein, fiber, and iron served as contributors to the association between the “Beans-vegetables” pattern and birth weight, which may explain the potential role of plant-based foods. It is worth mentioning the beneficial effect of plant protein in plant-based foods. There have been few studies that show a direct effect of maternal plant protein intake on birth weight. However, Lai et al. found a positive association between plant protein-enriched foods and optimal GWG (24). The results of another prospective study among adults who participated in the Diet, Genes, and Obesity (Diogenes) project also showed that the source but not the amount consumed was related to weight gain (29). Specifically, sources of protein that vary in amino acid composition should be considered here. Plant protein enhances insulin sensitivity and energy expenditure due to low in branched-chain amino acids (BCAAs) and sulfur-containing amino acids (SCAAs), which may interpret the association between plant protein and weight gain (30–32). Aside from plant protein, other characteristics of plant-based foods, including relatively low energy density and high fiber, merit attention. They play vital roles in appetite regulation, metabolism, and tissue maintenance in order to maintain a normal weight (24). On the contrary, animal-based foods are characterized as high cholesterol and saturated fatty acids (SFAs), both of which have been shown to be associated with an increased risk of obesity in previous studies (33, 34). Certainly, more strictly designed animal and human experiments are needed to further explore the specific mechanisms of the effect of plant-based food components on birth weight, either directly or indirectly *via* GWG.

The findings of the beneficial effect of the “Beans-vegetables” pattern only appeared in boys in the present study. This may be explained by a sex-specific growth mechanism during the fetal period. Generally, boys have higher birth weights, possibly due to an interaction between sex hormones, fetal insulin, and genetic factors (35). Available evidence suggested the sex-specific adaptation of the placenta may be the core of the differences in fetal growth (36). The placentas of male and female fetuses have different protein, gene expressions, instituting different mechanisms to cope with an adverse intrauterine environment or event, which is reflected in their birth weight (37–39). As for one of the focuses of intrauterine environment–maternal diet, it is proposed that boys are more sensitive and dependent on maternal diet during pregnancy, making them able to capitalize on improving food supply but vulnerable to food shortage (40, 41). It is because of this sensitive growth strategy of boys that warns of the need to pay more attention to intrauterine nutrition to improve birth outcomes.

The predominant advantage of this study is the use of prospective design to clarify the association between maternal dietary patterns and birth weight. Moreover, we took the mediation of GWG into consideration and analyzed direct and indirect effects of dietary patterns, respectively when exploring their relationship with birth weight. However, we acknowledge some limitations of this study. First, the dietary data of this study was obtained from a single FFQ investigation for 24–28 weeks. But as a previous study suggested, a single source of dietary data can provide reliable information throughout pregnancy (42). Second, using self-reported pre-pregnancy weight would introduce bias into GWG measurements. However, this is a practical and cost-effective method, and no matter in published literature or the sensitivity analysis of a previous study on the same population, the results revealed that the bias appeared to be small (43–45). Last, potential dietary and non-dietary confounders were adjusted in the analysis, while the possibility of residual confounding from unmeasured or unknown covariates could not be ruled out.

## Conclusions

In conclusion, this prospective cohort study in pregnant women in central China has shown that adherence to the “Beans-vegetables” pattern, which is characterized by high vegetables and beans intake, is beneficial for effectively controlling GWG in mothers and moderately increasing birth weight in newborns. In addition, GWG serves as a mediator in the association between this dietary pattern and birth weight, helping to control birth weight in a normal range. These novel findings may provide guidance for pregnant women to adhere to a healthy diet for beneficial pregnant outcomes. Well-designed intervention studies are needed to confirm our findings and elucidate the metabolic mechanisms underlying these findings.

## Data Availability Statement

The raw data supporting the conclusions of this article will be made available by the authors, without undue reservation.

## Ethics Statement

The studies involving human participants were reviewed and approved by Tongji Medical College, Huazhong University of Science and Technology. The patients/participants provided their written informed consent to participate in this study.

## Author Contributions

YL and XZ: conceived and designed the research, acquired the data, performed the statistical analysis, drafted the manuscript, and played an important role in interpreting the results. YZ, CZ, LHu, XC, RC, JW, QL, GS, HY, GX, LHa, and NY: acquired the data. XY: revised the manuscript and further contributed to the discussion. All authors read and approved the final vision of the manuscript.

## Funding

This work was supported by the National Program on Basic Research Project of China (NO.2013FY114200) for NY, and the National Natural Science Foundation of China (81973043) and Chinese Nutrition Society Nutrition Science Foundation—Feihe Tizhi, nutrition and health research fund (CNS-Feihe2019A33) for XY.

## Conflict of Interest

The authors declare that the research was conducted in the absence of any commercial or financial relationships that could be construed as a potential conflict of interest.

## Publisher's Note

All claims expressed in this article are solely those of the authors and do not necessarily represent those of their affiliated organizations, or those of the publisher, the editors and the reviewers. Any product that may be evaluated in this article, or claim that may be made by its manufacturer, is not guaranteed or endorsed by the publisher.
