# Supplementary material for: Association of Maternal Dietary Patterns With Birth Weight and the Mediation of Gestational Weight Gain: A Prospective Birth Cohort
Source: Front Nutr. 2021 Nov 26;8:782011. doi: 10.3389/fnut.2021.782011 (PMC8664542; doi:10.3389/fnut.2021.782011)
Supplement: Supplementary file 1 [file Table_1.pdf]

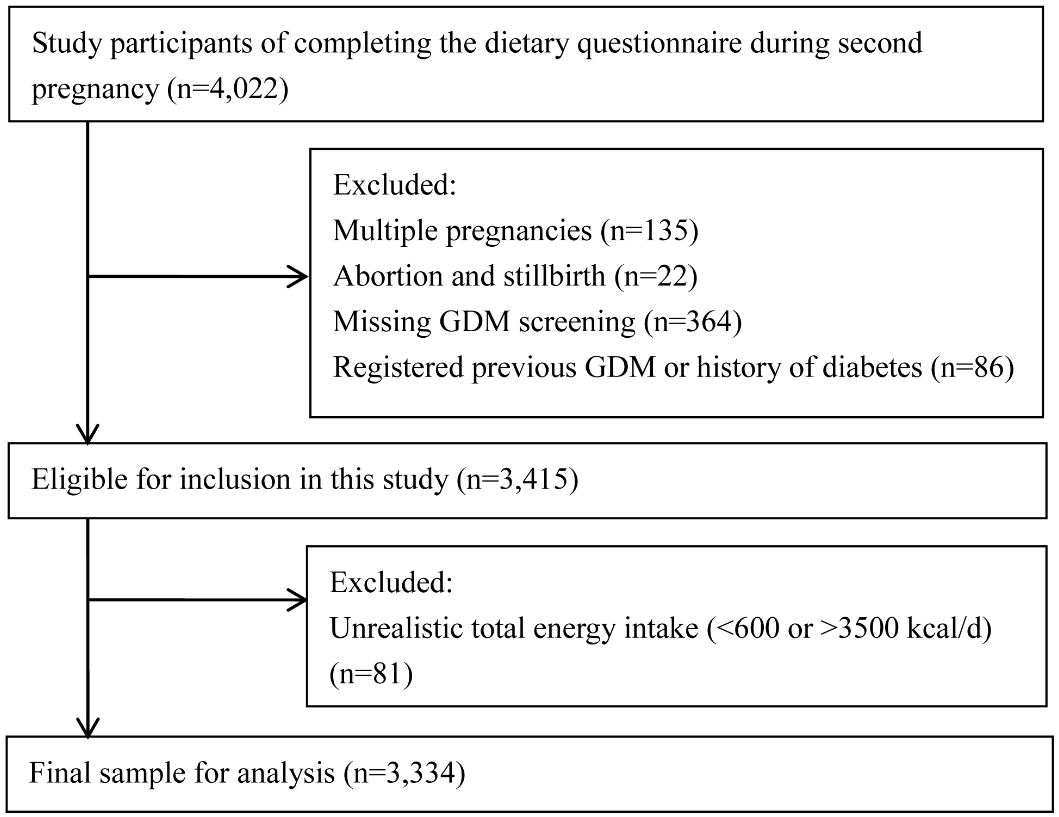

Supplement Figure 1 Flow diagram for selection of subjects for the study from the cohort

Supplement Table 1 Social and demographic characteristic of participants (n=3,334) [a].

Characteristic	This study
Age (y)	28.12±3.54
≤24	392 (11.8)
25-29	1985 (59.5)
30-35	831 (24.9)
≥36	126 (3.8)
Height (cm)	160.44±5.01
Pre-pregnancy BMI (kg/m^2)	20.77±2.71
<18.5	665 (20.0)
18.5-23.9	2277 (68.3)
24.0-27.9	335 (10.0)
⩾28.0	57 (1.7)
Parity	
0	2826 (84.8)
≥1	508 (15.2)
Ethnicity (Han Chinese)	3235 (97.0)
Education (schooling years)	
≤9	117 (3.5)
10-12	441 (13.2)
13-15	897 (26.9)
≥16	1794 (53.9)
Missing values	85 (2.5)
Average personal income (CNY/month)	
≤1000	15 (0.4)
1001-2999	249 (7.5)
3000-4999	1124 (33.7)
5000-9999	1327 (39.9)

≥10000	541 (16.2)
Missing values	78 (2.3)
Family history of diabetes (yes)	277 (8.3)
Family history of obesity (yes)	53 (1.6)
Smoking before pregnancy (yes)	120 (3.6)
Drinking before pregnancy (yes)	50 (1.5)
Physical activity (MET-hours/week)	28.35±21.34
Total energy intake (kcal/d)	1963.17±521.80

a Continuous variables were presented as mean ± SD (n), categorical variables were showed as n (%).

Supplement Table 2 Dietary pattern scores according to characteristics of participants (n=3,334) [a].

Variables	Number(%)	Dietary patterns				
		Beans-vegetables	Fish-meat-eggs	Nuts-whole grains	Organ-poultry-seafood	Rice-wheat-fruits
Age(y)						
≤24	392 (11.8)	-0.17 (0.99)	-0.16 (0.93)	-0.19 (0.97)	-0.01 (1.11)	0.14 (1.05)
25-29	1985 (59.5)	0.00 (0.99)	0.00 (0.98)	0.02 (0.99)	-0.03 (0.99)	0.02 (0.98)
30-35	831 (24.9)	0.09 (0.99)	0.04 (1.05)	0.05 (1.03)	0.06 (0.95)	-0.11 (1.05)
≥36	126 (3.8)	-0.12 (1.12)	0.26 (1.07)	-0.05 (0.95)	0.12 (1.04)	-0.07 (0.73)
P value [b]	—	0.001	<0.001	0.004	0.146	0.001
Ethnicity						
Han Chinese	3235 (97.0)	0.00 (1.00)	0.00 (1.00)	0.00 (1.00)	0.00 (1.00)	0.00 (1.01)
Others	99 (3.0)	0.18 (1.00)	0.00 (0.90)	-0.16 (1.02)	0.12 (0.96)	0.06 (0.62)
P value [b]	—	0.123	0.979	0.172	0.315	0.632
Education (schooling years)						
≤9	117 (3.5)	-0.39 (1.05)	-0.47 (1.04)	-0.39 (0.81)	0.05 (1.13)	0.03 (1.03)
10-12	441 (13.2)	-0.18 (0.98)	-0.30 (1.11)	-0.25 (0.97)	0.01 (1.10)	-0.07 (1.43)
13-15	897 (26.9)	-0.06 (1.05)	-0.12 (1.00)	0.00 (1.00)	-0.11 (1.04)	0.08 (0.80)
≥16	1794 (53.9)	0.09 (0.96)	0.13 (0.95)	0.07 (1.01)	0.05 (0.95)	-0.03 (0.98)
Missing values	85 (2.5)	-0.32 (1.00)	0.19 (0.87)	-0.09 (0.90)	-0.14 (0.97)	0.10 (0.87)
P value [b]	—	<0.001	<0.001	<0.001	0.005	0.087
Average personal income (CNY/month)						
≤1000	15 (0.4)	0.24 (0.83)	-0.27 (1.00)	0.02 (1.11)	-0.58 (1.06)	0.23 (0.41)
1001-2999	249 (7.5)	-0.09 (0.97)	-0.20 (1.06)	-0.05 (0.96)	-0.11 (1.11)	-0.09 (1.21)
3000-4999	1124 (33.7)	-0.12 (1.03)	-0.06 (1.00)	-0.03 (1.01)	-0.01 (0.98)	0.01 (1.09)
5000-9999	1327 (39.9)	0.05 (0.99)	0.05 (0.98)	-0.02 (0.99)	-0.01 (1.00)	-0.01 (0.97)
≥10000	541 (16.2)	0.15 (0.94)	0.06 (1.00)	0.12 (1.00)	0.08 (0.96)	0.04 (0.67)
Missing values	78 (2.3)	-0.41 (1.07)	0.00 (1.04)	-0.09 (1.15)	0.04 (1.08)	-0.12 (1.80)
P value [b]	—	<0.001	0.009	0.101	0.111	0.595
Family history of diabetes						
Yes	277 (8.3)	-0.04 (0.98)	0.11 (0.98)	-0.05 (1.04)	0.08 (1.07)	-0.24 (1.76)

No	3007 (90.2)	0.01 (1.00)	-0.01 (1.00)	0.01 (1.00)	0.00 (0.99)	0.02 (0.90)
Missing values	50 (1.5)	-0.20 (1.25)	-0.05 (0.86)	-0.06 (0.89)	-0.24 (1.05)	0.09 (0.65)
P value [b]	—	0.283	0.201	0.657	0.100	<0.001
Family history of obesity						
Yes	53 (1.6)	0.19 (0.95)	-0.18 (1.12)	0.03 (0.96)	-0.02 (1.09)	-0.11 (0.89)
No	3213 (96.4)	0.01 (1.00)	0.00 (1.00)	0.00 (1.00)	0.00 (1.00)	0.00 (1.01)
missing values	68 (2.0)	-0.37 (1.15)	-0.01 (0.92)	-0.21 (0.91)	-0.03 (1.08)	0.03 (0.83)
P value [b]	—	0.004	0.461	0.222	0.948	0.713
Smoking						
Yes	120 (3.6)	-0.11 (1.02)	-0.14 (1.03)	0.01 (1.05)	-0.04 (1.05)	0.00 (0.90)
No	3214 (96.4)	0.02 (0.99)	0.03 (0.99)	0.00 (0.99)	0.01 (0.99)	0.00 (1.02)
P value [b]	—	0.009	0.001	0.752	0.307	0.918
Drinking						
Yes	50 (1.5)	0.02 (0.94)	-0.11 (0.94)	0.10 (0.97)	-0.06 (1.02)	-0.04 (1.07)
No	3284 (98.5)	0.00 (1.01)	0.02 (1.01)	-0.02 (1.00)	0.01 (1.00)	0.01 (0.99)
P value [b]	—	0.592	0.012	0.024	0.156	0.403
Parity						
0	2826 (84.8)	-0.02 (1.00)	0.04 (0.98)	-0.01 (1.00)	-0.02 (1.00)	0.00 (1.04)
≥1	508 (15.2)	0.12 (0.99)	-0.20 (1.07)	0.04 (1.01)	0.09 (0.99)	-0.02 (0.77)
P value [b]	—	0.009	<0.001	0.428	0.055	0.661
Pre-pregnancy BMI(kg/m^2)						
<18.5	665 (20.0)	-0.06 (0.98)	0.03 (0.97)	0.06 (1.01)	-0.01 (1.02)	0.16 (0.65)
18.5-23.9	2277 (68.3)	0.02 (0.99)	-0.01 (0.99)	0.01 (1.00)	0.02 (0.97)	-0.02 (1.07)
24.0-27.9	335 (10.0)	0.00 (1.03)	0.02 (1.09)	-0.16 (0.97)	-0.10 (1.09)	-0.10 (1.03)
≥28.0	57 (1.7)	-0.05 (1.24)	-0.13 (1.03)	-0.20 (0.84)	0.03 (1.31)	-0.25 (0.72)
P value [b]	—	0.449	0.717	0.016	0.329	<0.001

a Dietary pattern scores created by multiplying factor loadings by corresponding standardized value for frequency intake of each food and adding all these items.

Values are mean factor scores, derived by extraction of five dietary factors. Not adjusted for confounders. Values are mean (SD) unless stated otherwise. BMI, body mass index.

b *P* values were from *t* test or ANOVA.

Supplement Table 3 Pregnant outcomes of all participants [a]

Pregnant outcomes	All participants
Maternal outcomes	
GWG (kg)	15.87±4.49
Insufficient GWG	21.8%
Adequate GWG	33.3%
Excessive GWG	44.9%
Gestational week of delivery (w)	39.30±1.64
GDM	11.5%
Newborn outcomes	
Birth weight (g)	3338.19±448.46
Normal birth weight (g)	3320.08±338.90
Birth length (cm)	50.14±1.50
PI (kg/m^3)	26.37±2.82
Sex	
Male	1665 (53.8)
Female	1427 (46.2)
Low birth weight	3.3%
Macrosomia	6.6%
SGA	7.3%
LGA	8.2%
Preterm	4.7%
Postmature	0.9%

a Continuous variables were presented as mean ± SD (n), categorical variables were showed as n (%). LGA, large for gestational age; SGA, small for gestational age; GDM, gestational diabetes mellitus; GWG, gestational weight gain; PI, ponderal index.

Supplement Table 4 Contributions of nutrients in the association between "Beans-vegetables" patterns and normal birth weight (g) [a].

Variable	Quartiles of "Beans-vegetables" pattern scores			
	Q1	Q2	Q3	Q4
Total				
Adjusted model + total protein	1 (Reference)	10.42 (-24.71 ,45.55) [b]	7.43 (-28.45 ,43.31)	37.89 (1.08 ,74.70)
Adjusted model + animal protein	1 (Reference)	9.90 (-25.31 ,45.11)	6.48 (-29.68 ,42.65)	37.40 (0.21 ,74.58)
Adjusted model + plant protein	1 (Reference)	14.08 (-20.99 ,49.15)	14.99 (-20.59 ,50.56)	51.14 (15.41 ,86.87)
Adjusted model + total fat	1 (Reference)	12.77 (-22.25 ,47.80)	11.94 (-23.53 ,47.41)	45.73 (10.12 ,81.34)
Adjusted model + animal fat	1 (Reference)	12.50 (-22.53 ,47.52)	11.65 (-23.76 ,47.07)	44.60 (9.08 ,80.11)
Adjusted model + plant fat	1 (Reference)	13.12 (-21.90 ,48.13)	11.92 (-23.46 ,47.3)	45.07 (9.76 ,80.38)
Adjusted model + carbohydrate	1 (Reference)	11.28 (-23.78 ,46.34)	8.56 (-27.11 ,44.22)	41.09 (5.25 ,76.94)
Adjusted model + fiber	1 (Reference)	11.02 (-24.22 ,46.27)	8.22 (-28.33 ,44.77)	39.85 (1.46 ,78.24)
Adjusted model + cholesterol	1 (Reference)	12.99 (-22.03 ,48.02)	12.60 (-22.78 ,47.99)	47.15 (11.93 ,82.37)
Adjusted model + vitamin A	1 (Reference)	11.73 (-23.34 ,46.79)	9.54 (-26.16 ,45.23)	42.20 (6.18 ,78.22)
Adjusted model + retinol	1 (Reference)	13.10 (-21.92 ,48.12)	12.50 (-22.87 ,47.87)	47.10 (11.94 ,82.25)
Adjusted model + carotene	1 (Reference)	11.67 (-23.44 ,46.78)	9.77 (-26.08 ,45.61)	42.53 (6.08 ,78.97)
Adjusted model + vitamin E	1 (Reference)	12.64 (-22.34 ,47.63)	12.74 (-22.59 ,48.07)	47.50 (12.38 ,82.61)
Adjusted model + vitamin C	1 (Reference)	11.43 (-23.69 ,46.56)	8.70 (-27.42 ,44.81)	41.09 (4.13 ,78.04)
Adjusted model + iron	1 (Reference)	10.06 (-25.12 ,45.24)	6.71 (-29.38 ,42.79)	37.39 (0.26 ,74.52)
Adjusted model + heme iron	1 (Reference)	12.66 (-22.35 ,47.68)	11.67 (-23.72 ,47.07)	44.79 (9.43 ,80.16)
Adjusted model + non-heme iron	1 (Reference)	10.18 (-25.03 ,45.40)	7.11 (-29.07 ,43.29)	38.35 (1.12 ,75.59)
Boys				
Adjusted model + total protein	1 (Reference)	0.80 (-48.72 ,50.31)	10.39 (-39.44 ,60.22)	53.42 (2.65 ,104.20)
Adjusted model + animal protein	1 (Reference)	0.82 (-48.46 ,50.10)	11.27 (-37.87 ,60.40)	52.90 (3.13 ,102.66)
Adjusted model + plant protein	1 (Reference)	-0.36 (-50.16 ,49.45)	8.87 (-41.47 ,59.21)	50.66 (-1.72 ,103.04)
Adjusted model + total fat	1 (Reference)	2.01 (-47.25 ,51.27)	12.23 (-37.00 ,61.46)	55.96 (6.14 ,105.78)
Adjusted model + animal fat	1 (Reference)	1.19 (-47.99 ,50.37)	11.31 (-37.76 ,60.37)	52.84 (3.51 ,102.18)
Adjusted model + plant fat	1 (Reference)	5.64 (-43.58 ,54.86)	18.75 (-30.59 ,68.09)	66.56 (16.73 ,116.39)
Adjusted model + carbohydrate	1 (Reference)	-0.39 (-49.82 ,49.05)	9.12 (-40.39 ,58.63)	50.86 (0.52 ,101.19)
Adjusted model + fiber	1 (Reference)	0.52 (-49.14 ,50.17)	9.24 (-41.58 ,60.05)	50.88 (-3.36 ,105.11)
Adjusted model + cholesterol	1 (Reference)	2.01 (-47.20 ,51.23)	12.45 (-36.62 ,61.51)	56.11 (6.86 ,105.35)
Adjusted model + vitamin A	1 (Reference)	0.67 (-48.65 ,49.99)	9.50 (-40.11 ,59.11)	51.48 (0.86 ,102.11)
Adjusted model + retinol	1 (Reference)	1.27 (-47.94 ,50.47)	11.73 (-37.34 ,60.80)	55.12 (6.03 ,104.22)
Adjusted model + carotene	1 (Reference)	1.58 (-47.74 ,50.90)	11.02 (-38.78 ,60.83)	54.12 (3.04 ,105.20)
Adjusted model + vitamin E	1 (Reference)	3.85 (-45.54 ,53.25)	15.67 (-34.17 ,65.51)	62.16 (10.75 ,113.58)
Adjusted model + vitamin C	1 (Reference)	1.68 (-47.60 ,50.97)	10.84 (-39.22 ,60.89)	53.74 (1.85 ,105.63)
Adjusted model + iron	1 (Reference)	-1.37 (-51.07 ,48.34)	7.40 (-42.75 ,57.55)	47.65 (-4.68 ,99.98)
Adjusted model + heme iron	1 (Reference)	0.47 (-48.80 ,49.73)	10.94 (-38.18 ,60.07)	52.41 (2.81 ,102.01)
Adjusted model + non-heme iron	1 (Reference)	-0.50 (-50.20 ,49.20)	8.45 (-41.79 ,58.69)	49.79 (-2.50 ,102.09)

a Multivariate linear regression models were used. Adjusted model was adjusted for other dietary patterns, maternal age, physical activity, ethnology, maternal education, average personal income, family history of diabetes, family history of obesity, smoking habit, alcohol habit, parity, pre-pregnancy BMI, GDM, gestational weight gain, and total energy intake.

b Values were presented as β (95% *CI*) (all such values).
